# Tumor-specific HMG-CoA reductase expression in primary premenopausal breast cancer predicts response to tamoxifen

**DOI:** 10.1186/bcr2820

**Published:** 2011-01-31

**Authors:** Donal J Brennan, Henriette Laursen, Darran P O'Connor, Signe Borgquist, Mathias Uhlen, William M Gallagher, Fredrik Pontén, Robert C Millikan, Lisa Rydén, Karin Jirström

**Affiliations:** 1 UCD School of Biomolecular and Biomedical Science, UCD Conway Institute, University College Dublin, Belfield, Dublin 4, Ireland; 2 Department of Oncology, Clinical Sciences, Lund University, Skåne University Hospital, 221 85 Lund, Sweden; 3 Department of Biotechnology, AlbaNova University Center, Royal Institute of Technology, 106 91 Stockholm, Sweden; 4 Department of Genetics and Pathology, Rudbeck Laboratory, Uppsala University, 751 85 Uppsala, Sweden; 5 Department of Epidemiology, University of North Carolina at Chapel Hill, Chapel Hill, NC 27599, USA; 6 Department of Surgery, Clinical Sciences, Lund University, Skåne University Hospital, 221 85 Lund, Sweden; 7 Department of Pathology, Clinical Sciences, Lund University, Skåne University Hospital, 221 85 Lund, Sweden

## Abstract

**Introduction:**

We previously reported an association between tumor-specific 3-hydroxy-3-methylglutharyl-coenzyme A reductase (HMG-CoAR) expression and a good prognosis in breast cancer. Here, the predictive value of HMG-CoAR expression in relation to tamoxifen response was examined.

**Methods:**

HMG-CoAR protein and RNA expression was analyzed in a cell line model of tamoxifen resistance using western blotting and PCR. HMG-CoAR mRNA expression was examined in 155 tamoxifen-treated breast tumors obtained from a previously published gene expression study (Cohort I). HMG-CoAR protein expression was examined in 422 stage II premenopausal breast cancer patients, who had previously participated in a randomized control trial comparing 2 years of tamoxifen with no systemic adjuvant treatment (Cohort II). Kaplan-Meier analysis and Cox proportional hazards modeling were used to estimate the risk of recurrence-free survival (RFS) and the effect of HMG-CoAR expression on tamoxifen response.

**Results:**

HMG-CoAR protein and RNA expression were decreased in tamoxifen-resistant MCF7-LCC9 cells compared with their tamoxifen-sensitive parental cell line. HMG-CoAR mRNA expression was decreased in tumors that recurred following tamoxifen treatment (*P *< 0.001) and was an independent predictor of RFS in Cohort I (hazard ratio = 0.63, *P *= 0.009). In Cohort II, adjuvant tamoxifen increased RFS in HMG-CoAR-positive tumors (*P *= 0.008). Multivariate Cox regression analysis demonstrated that HMG-CoAR was an independent predictor of improved RFS in Cohort II (hazard ratio = 0.67, *P *= 0.010), and subset analysis revealed that this was maintained in estrogen receptor (ER)-positive patients (hazard ratio = 0.65, *P *= 0.029). Multivariate interaction analysis demonstrated a difference in tamoxifen efficacy relative to HMG-CoAR expression (*P *= 0.05). Analysis of tamoxifen response revealed that patients with ER-positive/HMG-CoAR tumors had a significant response to tamoxifen (*P *= 0.010) as well as patients with ER-positive or HMG-CoAR-positive tumors (*P *= 0.035). Stratification according to ER and HMG-CoAR status demonstrated that ER-positive/HMG-CoAR-positive tumors had an improved RFS compared with ER-positive/HMG-CoAR-negative tumors in the treatment arm (*P *= 0.033); this effect was lost in the control arm (*P *= 0.138), however, suggesting that HMG-CoAR predicts tamoxifen response.

**Conclusions:**

HMG-CoAR expression is a predictor of response to tamoxifen in both ER-positive and ER-negative disease. Premenopausal patients with tumors that express ER or HMG-CoAR respond to adjuvant tamoxifen.

## Introduction

3-Hydroxy-3-methylglutharyl-coenzyme A reductase (HMG-CoAR) acts as a rate-limiting enzyme in the mevalonate pathway. The main product of the mevalonate pathway is cholesterol; however, the pathway also produces a number of nonsterol isoprenoid side products, which are important regulators of angiogenesis, proliferation, and migration [1,2]. HMG-CoAR inhibitors (statins) have demonstrated anti-neoplastic effects *in vitro *[3-5] and in xenograft models [5]. Statins have been suggested to lower the cancer incidence [6], but to date epidemiological studies have failed to confirm an association between statin use and overall breast cancer risk [7-10]. A lower incidence of estrogen receptor (ER)-negative tumors has, however, been reported among statin users [11]. Furthermore, an inverse relationship between postdiagnosis statin use and breast cancer recurrence has been reported [12].

We previously demonstrated an association between tumor-specific HMG-CoAR expression and improved prognosis in both breast cancer and epithelial ovarian cancer [13-15]. Using immunohistochemistry in 511 incident breast cancer cases within the population-based prospective cohort Malmö Diet and Cancer Study [16], we demonstrated that increased levels of HMG-CoAR protein expression were associated with favorable characteristics such as a smaller tumor size, low histological grade and ER positivity [13]. A validation study confirmed these findings and demonstrated that HMG-CoAR was an independent prognostic marker, associated with an improved recurrence-free survival (RFS) that was particularly evident in ER-positive tumors [14].

Based on these findings we sought to investigate the predictive value HMG-CoAR expression in tamoxifen-treated breast cancer patients. The relationship between HMG-CoAR expression and tamoxifen response was initially examined *in vitro *using a cell line model of tamoxifen resistance [17]. HMG-CoAR mRNA expression was then examined in a gene expression dataset published by Chanrion and colleagues containing 155 primary breast tumors obtained from patients treated with 5 years of adjuvant tamoxifen [18]. Finally HMG-CoAR protein expression was examined in premenopausal patients with stage II (pT2 N0 M0, pT1-2 N1 M0) invasive breast cancer. These patients had participated in a prospective randomized trial for 2 years of adjuvant tamoxifen versus no systemic treatment [19].

## Materials and methods

### Cell lines

MCF-7 cells and their tamoxifen-resistant derivative LCC9 were obtained from Prof. Robert Clarke (Georgetown University, Washington, DC, USA) and were maintained as previously described [17].

### Western blotting

Western blot analysis was performed as previously described [20]. The primary antibody used was a polyclonal anti-HMG-CoAR antibody (Upstate catalog number 07-457; Millipore, Temecula, CA, USA) diluted 1:500 (2 μg/ml). An anti-β-actin antibody (Clone 8226; Abcam, Cambridge, UK) at a dilution of 1:5000 was used as a loading control.

### Cell pellet arrays

Cell lines were fixed in 4% formalin and processed in gradient alcohols. Cell pellets were cleared in xylene and washed multiple times in molten paraffin. Once processed, cell lines were arrayed in duplicate 1.0 mm cores using a manual tissue arrayer (MTA-1; Beecher Instruments Inc., Sun Prairie, WI, USA).

### Quantitative SYBR Green real-time PCR

Total RNA was isolated from cell lines using Trizol (Invitrogen, Carlsbad, CA, USA) and reverse-transcribed using SuperScript II™ Reverse Transcriptase (Invitrogen) according to the manufacturer's instructions. HMG-CoAR-F (5'-GGACCCCTTTGCTTAGATGAAA-3') and HMG-CoAR-R (5'-CCACCAAGACCTATTGCTCTG-3') primers were designed using Primer Express software (Version 2.0; Applied Biosystems, Warrington, UK) and were used to amplify a HMG-CoAR-specific DNA fragment with SYBR Green PCR Master Mix (Applied Biosystems) using a 7900HT Fast Real-Time PCR System (Applied Biosystems). Relative HMG-CoAR expression levels in untreated MCF-7 cells versus LCC9 cells were calculated using the qBase real-time PCR relative quantification software [21], with all samples normalized to 18s rRNA. Negative controls included a no-template control and a no-reverse-transcriptase control. All quantitative reverse transcriptase PCR reactions were performed in triplicate.

### MTT assay

The tamoxifen response in MCF-7 and LCC9 cells was measured using a 3-(4,5-dimethylthiazol-2-yl)-2,5-diphenyltetrazolium bromide (MTT) assay. A sample of 10^5 ^cells was plated in 96-well plates and grown for 48 hours. Fresh medium containing various concentrations of 4-hydroxy-tamoxifen (Sigma, Saint Louis, MO, USA) was added to each well and cells were incubated for 5 days. On day 5, 50 μl of 5 mg/ml MTT (Sigma) in PBS were added to each well. After 4 hours at 37°C, the medium was carefully removed from the wells and the remaining formazan crystals were dissolved in dimethylsulfoxide. The absorbance at 570 nm was read on a microplate reader.

### Patients

Cohort I consisted of a gene expression dataset published by Chanrion and colleagues [18], containing 155 primary breast tumors obtained from patients who had undergone initial surgery between 1989 and 2001 at the Cancer Research Center of Val d'Aurelle in Montpellier, the Bergonié Institute in Bordeaux, or the Department of Obstetrics and Gynecology of Turin. The median follow-up time for all patients was 5.5 years. The aim of this study was to identify a gene expression signature associated with tamoxifen resistance. Eight tumors were ER-negative, and six of these tumors were progesterone receptor (PR)-positive. No patient received neoadjuvant or adjuvant systemic chemotherapy as first-line therapy. All patients were treated with adjuvant tamoxifen (20 mg daily) for 5 years. One hundred and twenty-one patients also received adjuvant radiotherapy. Recurrence was observed in 52 patients (48 distant metastases and four local recurrences) with a median relapse time of 37.1 months. Raw gene expression data and clinical data were downloaded from Gene Expression Omnibus [GEO:GSE9893] [22]. The log ratio of gene expression values was used without further transformation. For statistical analysis, HMG-CoAR expression levels were analyzed as a continuous variable.

Cohort II consisted of 564 premenopausal women with primary breast cancer in the south and southeast regions of Sweden were enrolled in a multicenter clinical trial and were randomized to either 2 years of adjuvant tamoxifen (*n *= 276) or a control group (*n *= 288) irrespective of hormonal receptor status [19]. The aim of this study was to examine the effect of tamoxifen on RFS, and the study has been described in detail elsewhere [19]. RFS was considered local, regional, distant recurrences and breast cancer-specific death, but not contralateral breast cancer. The inclusion criteria were premenopausal patients, or patients younger than 50 years, with stage II (pT2 N0 M0, pT1-2 N1 M0) invasive breast cancer treated by modified radical mastectomy or breast-conserving surgery with axillary lymph node dissection. Postoperative radiotherapy (50 Gy) was administered after breast-conserving surgery, and all lymph-node-positive patients received locoregional radiotherapy. Less than 2% of the patients received adjuvant systemic chemotherapy. The median follow-up time for patients without breast cancer events was 13.9 years. The ethics committees at Lund and Linköping Universities approved the study. Oral informed consent was registered for all patients. The results of the trial have been previously described [19] and the trial has been included in the Oxford meta-analysis [23].

### Tissue microarray construction

Five hundred paraffin-embedded tumor specimens were used for tissue microarray (TMA) construction. TMAs were constructed as described previously [24]. In brief, two 0.6 mm cores were taken from areas representative of invasive cancer and were mounted in a recipient block using a manual arraying device (MTA-1; Beecher Inc.). The study was approved by the ethics committees at Linköping University and Lund University.

### Immunohistochemistry

Four-micometer sections from the TMAs and 3.5 μm sections from the cell pellet arrays were automatically pretreated using the PT-link system (DAKO, Copenhagen, Denmark) and were then stained in a Techmate 500 (DAKO) with a polyclonal anti-HMG-CoAR antibody (Upstate catalog number 07-457) diluted 1:250.

For all other antibodies, heat-mediated antigen retrieval was performed using microwave treatment for 2 × 5 minutes in a citrate buffer before being processed either in the Ventana Benchmark system (Ventana Medical Systems Inc., Tucson, AZ, USA) using pre-diluted antibodies to ER (anti-ER, clone 6F11), PR (anti-PgR, clone 16) and Her2 (Pathway CB-USA, 760-2694) or in the Techmate 500 (DAKO) for Ki-67 (1:200, M7240; DAKO).

Cytoplasmic staining of HMG-CoAR was assessed by two investigators, one of whom is a board-certified pathologist (KJ), according to intensity (negative = 0, weak = 1, moderate = 2, strong = 3). HMG-CoAR was not expressed in the nucleus. Discordant cores were reassessed jointly and a consensus was reached. HER2 and Ki-67 were assessed as previously described [20]. ER-negativity and PR-negativity was defined as <10% positively staining nuclei, according to current clinical guidelines in Sweden.

### Statistical analysis

Differences in distribution of clinical data and tumor characteristics between HMG-CoAR-negative and HMG-CoAR-positive tumors were evaluated using the chi-square test. Kaplan-Meier analysis and the log-rank test were used to illustrate differences between RFS according to HMG-CoAR expression. Cox regression proportional hazards models were used to estimate the impact of HMG-CoAR expression on RFS and overall survival (OS) in both univariate and multivariate analysis, adjusted for tumor size, age at diagnosis, ER status, HER2 status, lymph node status and Nottingham Histological Grade in the entire cohort. Cox proportional hazards models were used to estimate relative hazards adjusted or not for potential prognostic factors. The model was used in Cohort II to estimate the interaction effect between tamoxifen treatment and HMG-CoAR expression in order to measure any possible difference in treatment effect based on HMG-CoAR expression. For this purpose an interaction variable was constructed: TAM treatment (+/-) × HMG-COAR (+/-). All calculations were performed using SPSS version 15.0 (SPSS Inc., Chicago, IL, USA). All statistical tests were two-sided and *P *< 0.05 was considered statistically significant.

## Results

### HMG-CoAR is associated with tamoxifen response *in vitro*

**Figure 1:**
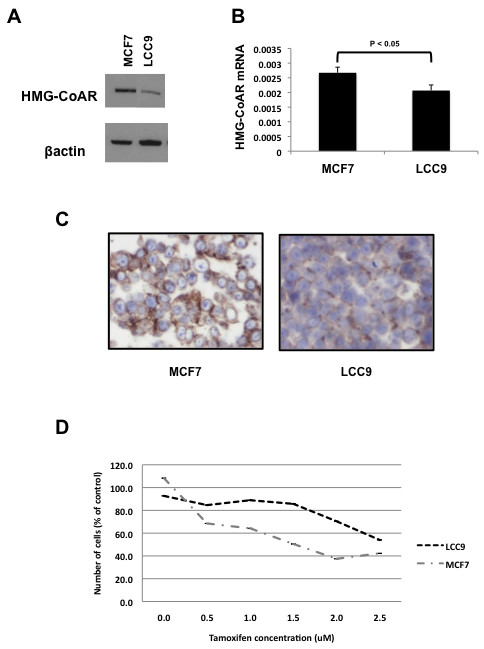
**HMG-CoAR expression is associated with tamoxifen response *in vitro***. **(a) **Western blot demonstrating increased expression of 3-hydroxy-3-methylglutharyl-coenzyme A reductase (HMG-CoAR) in tamoxifen-sensitive MCF7 cells compared with their tamoxifen-resistant derivatives LCC9. **(b) **Quantitative reverse transcriptase PCR demonstrating increased expression of HMG-CoAR mRNA in MCF7 cells compared with LCC9 cells. **(c) **Immunohistochemistry demonstrating increased HMG-CoAR protein expression in MCF7 cells compared with LCC9 cells. **(d) **MTT assay demonstrating improved tamoxifen response in MCF7 cells compared with LCC9 cells.

The specificity of the anti HMG-CoAR antibody was confirmed in a previous study [13]. The anti-HMG-CoAR antibody recognized a single distinct band at ~90 kDa in MCF7 cells. HMG-CoAR protein expression was significantly decreased in the tamoxifen-resistant derivative LCC9 cell line (Figure 1a). HMG-CoAR mRNA expression was also decreased in the LCC9 cells compared with their MCF7 derivatives (Figure 1b). Immunohistochemistry performed on the same cell lines confirmed these results (Figure 1c). Finally treatment of the cell lines with tamoxifen demonstrated anti-estrogen resistance in the LCC9 cells compared with their MCF7 derivatives (Figure 1d).

### HMG-CoAR mRNA expression is associated with a prolonged recurrence-free survival in tamoxifen-treated breast cancer patients

**Figure 2:**
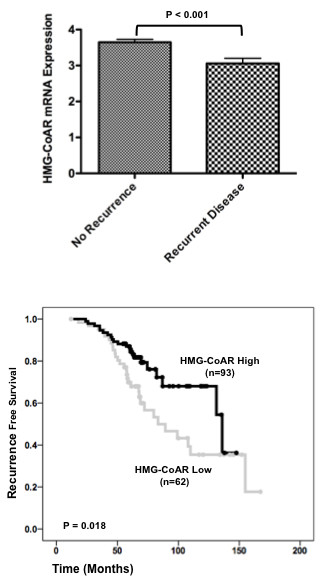
**HMG-CoAR mRNA expression is associated with increased recurrence-free survival in tamoxifen-treated breast cancer patients**. **(a) **Mean 3-hydroxy-3-methylglutharyl-coenzyme A reductase (HMG-CoAR) mRNA levels were significantly higher in patients who remained disease-free after tamoxifen treatment. *P < 0.001. **(b) **Kaplan-Meier using estimates of recurrence-free survival according to HMG-CoAR mRNA expression using mean HMG-CoAR expression as a threshold.

The relationship between HMG-CoAR mRNA expression and tamoxifen response was examined in Cohort I. Chanrion and colleagues' dataset consists of patients treated with 5 years of adjuvant tamoxifen and contains both premenopausal and postmenopausal patients [18]. HMG-CoAR mRNA levels were higher in patients who remained disease-free following tamoxifen treatment compared with those who developed recurrences (Figure 2a). Using a threshold of mean expression, Kaplan-Meier analysis demonstrated that increased HMG-CoAR mRNA expression was associated with a prolonged RFS (*P *= 0.018) (Figure 2b).

Examination of the relationship between HMG-CoAR mRNA expression and other clinicopathological parameters revealed that increased HMG-CoAR mRNA expression was associated with small (*P *= 0.003), lymph-node-negative (*P *= 0.003) tumors (Table 1). Univariate analysis of HMG-CoAR as a continuous variable revealed that increased levels of HMG-CoAR mRNA were associated with a prolonged RFS and OS in Cohort I (Table 2). Multivariate Cox regression analysis controlling for grade, age, nodal status and tumor size confirmed that HMG-CoAR was an independent predictor of RFS (hazard ratio = 0.63, 95% confidence interval = 0.45 to 0.89, *P *= 0.009) and OS (hazard ratio = 0.49, 95% confidence interval = 0.32 to 0.75, *P *= 0.001) (Table 2), suggesting that HMG-CoAR may play an important role in tamoxifen-treated breast cancer patients.

**Table 1:** Clinical and tumor characteristics stratified according to HMG-CoAR protein expression in two independent cohorts

	Cohort I			Cohort II		
		
	Low HMG-CoAR (%) (*n *= 62)	High HMG-CoAR (%) (*n *= 93)	***P *value (χ**^**2 **^**test)**	HMG-CoAR-negative (%) (*n *= 222)	HMG-CoAR-positive (%) (*n *= 200)	***P *value (χ**^**2 **^**test)**
Age (years)						
<median	34 (55)	42 (45)	0.238	36 (16)	36 (18)	0.612
>median	28 (45)	51 (55)		186 (84)	164 (82)	
Tumor size						
0 to 20 mm	21 (35)	57 (63)	0.003	75 (34)	79 (40)	0.196
>21 mm	39 (65)	33 (37)		147 (66)	120 (60)	
Missing	5					
Histological subtype						
Ductal	49 (79)	75 (81)	0.250	181 (91)	171 (93)	0.581
Others	13 (21)	18 (19)		17 (9)	13 (7)	
Unknown				40		
Nodal status						
N0	21 (35)	57 (63)	0.003	70 (32)	42 (21)	0.009
N1+	39 (65)	33 (37)		152 (68)	158 (79)	
Unknown	5					
Nottingham Histological Grade						
I	9 (15)	12 (13)	0.535	15 (7)	28 (15)	0.009
II	38 (60)	56 (60)		85 (40)	86 (45)	
III	10 (14)	23 (27)		115 (53)	79 (40)	
Unknown	7			14		
ER status						
Negative				80 (38)	54 (28)	0.026
Positive				130 (62)	141 (72)	
Unknown				17		
PR status						
Negative				78 (38)	56 (30)	0.098
Positive				130 (62)	133 (70)	
Unknown				25		
Ki-67						
<10%				138 (73)	128 (71)	0.658
>10%				52 (27)	53 (29)	
Unknown				51		
Her2 immunohistochemistry						
0 to 2+				173 (90)	137 (80)	0.008
3+				20 (10)	35 (20)	
Unknown				57		

Data in parenthesis are percentages unless otherwise stated. ER, estrogen receptor; HMG-CoAR, 3-hydroxy-3-methylglutharyl-coenzyme A reductase; N0, node-negative; N1+, node-positive; PR, progesterone receptor.

**Table 2:** Cox analysis of recurrence-free and overall survival according to HMG-CoAR mRNA expression in Cohort I

	Recurrence-free survival						Overall survival					
		
	Univariate			**Multivariate**^**a**^			Univariate			**Multivariate**^**a**^		
				
	HR	95% CI	*P *value	HR	95% CI	*P *value	HR	95% CI	*P *value	HR	95% CI	*P *value
HMG-CoAR mRNA (continuous)	0.64	0.48 to 0.85	0.002	0.63	0.45 to 0.89	0.009	0.55	0.41 to 0.75	<0.001	0.49	0.32 to 0.75	0.001
Size (continuous)	1.02	0.99 to 1.05	0.175	1.02	0.97 to 1.07	0.439	0.99	0.96 to 1.04	0.89	0.98	0.93 to 1.04	0.581
Grade (I and II vs. III)	2.62	1.62 to 4.23	<0.001	3.13	1.80 to 5.44	<0.001	2.95	1.70 to 5.14	<0.001	4.32	2.22 to 8.41	<0.001
Nodal status (negative vs. positive)	2.80	1.44 to 5.42	0.002	1.13	1.10 to 1.21	<0.001	5.49	2.29-13.17	<0.001	1.12	1.04 to 1.20	0.004
Age (continuous)	1.02	0.99 to 1.04	0.23	0.99	0.96 to 1.03	0.700	1.01	0.98 to 1.04	0.599	0.99	0.95 to 1.03	0.566

CI, confidence interval; HMG-CoAR, 3-hydroxy-3-methylglutharyl-coenzyme A reductase; HR, hazard ratio. ^a^Adjusted for all other variables in the table.

### HMG-CoAR protein expression predicts tamoxifen response in premenopausal breast cancer

Having demonstrated a relationship between HMG-CoAR mRNA expression in tamoxifen-treated patients, we proceeded to examine HMG-CoAR protein expression in Cohort II. Tumor samples were available from 500 of 564 patients (89%) included in the randomized study. Following antibody optimization and staining, it was possible to evaluate the expression of HMG-CoAR protein in 422 (84.4%) of the 500 tumors represented on the TMA.

**Figure 3:**
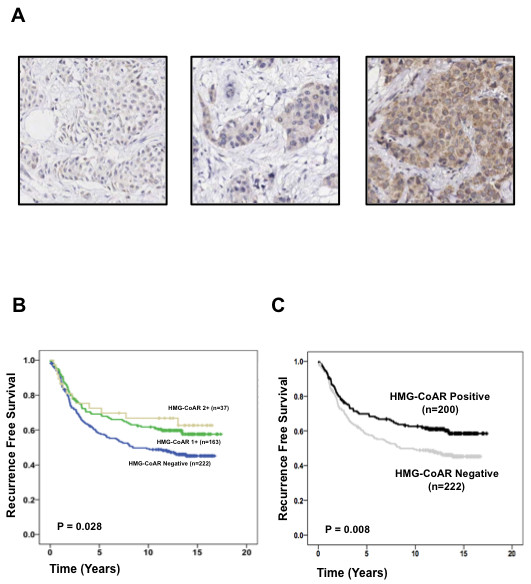
**HMG-CoAR protein expression in Cohort II**. **(a) **Immunohistochemical analysis of 3-hydroxy-3-methylglutharyl-coenzyme A reductase (HMG-CoAR) demonstrating different levels of staining intensity. **(b) **Kaplan-Meier estimates of recurrence-free survival according to HMG-CoAR expression in three groups. **(c) **Kaplan-Meier estimates of recurrence-free survival according to HMG-CoAR expression (negative = 0 or positive = 1 to 3) in all patients (*n *= 422).

Only staining intensity was accounted for in the statistical analyses of HMG-CoAR protein expression, as HMG-CoAR, when present, was generally expressed in the majority of tumor cells (>50%) - a finding consistent with previous studies [13,14]. Two hundred and twenty-three (52.7%) tumors lacked HMG-CoAR expression, 163 (38.5%) demonstrated a weak signal, 37 (8.7%) a moderate signal and none demonstrated a strong signal. Examples of HMG-CoAR expression are illustrated in Figure 3a.

To validate our previous findings [13,14], and also to identify any differences in HMG-CoAR expression in an exclusively premenopausal cohort, the relationship between HMG-CoAR protein expression and established clinicopathological variables was evaluated. As demonstrated in Table 1, HMG-CoAR expression, dichotomized to absent staining versus any staining, was associated with low histological grade (*P *= 0.009), ER-positivity (*P *= 0.026), and Her2 overexpression (*P *= 0.008). HMG-CoAR protein expression was associated with lymph-node-positivity in Cohort II (*P *= 0.009).

Assessment of the evaluable tumors in Cohort II (*n *= 422) revealed that HMG-CoAR protein expression was associated with a stepwise increased RFS (*P *= 0.028) when analyzed in three groups (negative, 1+ and 2+) (Figure 3b). Dichotomization of HMG-CoAR protein expression data to absent expression versus any expression demonstrated an association with an improved RFS (*P *= 0.008) (Figure 3c). HMG-CoAR was also associated with an improved breast-cancer-specific survival and OS (data not shown). As the aim of the original trial was to examine the effect of tamoxifen on RFS, this was used as the endpoint in this study. As illustrated in Table 3, multivariate Cox regression analysis revealed that HMG-CoAR expression was an independent positive prognostic factor in the evaluated cohort (hazard ratio = 0.67, 95% confidence interval = 0.49 to 0.91, *P *= 0.010).

**Table 3:** Cox analysis of recurrence-free survival according to HMG-CoAR protein expression in Cohort II

	All tumors (*n *= 422)						ER-positive (*n *= 270)					
		
	Univariate			**Multivariate**^**a**^			Univariate			**Multivariate**^**a**^		
				
	HR	95% CI	*P *value	HR	95% CI	*P *value	HR	95% CI	*P *value	HR	95% CI	*P *value
HMG-CoAR (0 vs. 1 to 2+)	0.68	0.51 to 0.90	0.009	0.67	0.49 to 0.91	0.01	0.66	0.46 to 0.95	0.024	0.65	0.45 to 0.96	0.029
Grade (I and II vs. III)	1.88	1.46 to 2.42	<0.001	1.58	1.11 to 2.25	0.012	1.85	1.32 to 2.60	<0.001	1.56	1.04 to 2.34	0.03
Age (continuous)	0.95	0.95 to 0.99	0.002	0.97	0.95 to 1.01	0.071	0.95	0.93 to 0.98	0.001	0.95	0.92 to 0.99	0.015
Nodal status (negative vs. positive)	1.7	1.26 to 2.29	0.001	2.12	1.47 to 3.28	<0.001	1.42	0.94 to 2.15	0.098	1.56	0.95 to 2.57	0.079
Tumor size (continuous)	1.16	0.90 to 1.50	0.254	1.27	0.91 to 1.77	0.159	1.17	0.84 to 1.64	0.361	1.22	0.81 to 1.84	0.352
ER status (0 to 10% vs. 11 to 100%)	0.71	0.54 to 0.94	0.018	0.65	0.37 to 1.13	0.126						
PR status (0 to 10% vs. 11 to 100%)	0.73	0.54 to 0.97	0.031	1.37	0.77 to 2.43	0.285	0.94	0.832 to 1.19	0.95	2.28	0.72 to 7.27	0.163
Treatment (tamoxifen vs. control)	0.78	0.60 to 0.98	0.033	0.68	0.50 to 0.92	0.011	0.61	0.44 to 0.86	0.005	0.58	0.39 to 0.84	0.005

CI, confidence interval; ER, estrogen receptor; HMG-CoAR, 3-hydroxy-3-methylglutharyl-coenzyme A reductase; HR, hazard ratio; PR, progesterone receptor. ^a^Adjusted for all other variables in the table.

**Figure 4:**
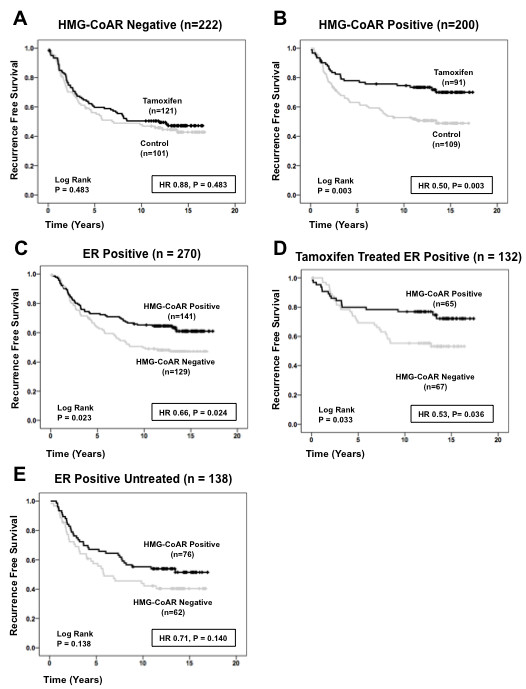
**HMG-CoAR protein expression is associated with tamoxifen response in Cohort II**. **(a) **Kaplan-Meier estimate of recurrence-free survival comparing 2 years of tamoxifen treatment with no adjuvant treatment in 3-hydroxy-3-methylglutharyl-coenzyme A reductase (HMG-CoAR)-negative tumors. **(b) **Kaplan-Meier estimate of recurrence-free survival comparing 2 years of tamoxifen treatment with no adjuvant treatment in HMG-CoAR-positive tumors. **(c) **Kaplan-Meier estimate of recurrence-free survival in all estrogen receptor (ER)-positive tumors. **(d) **Kaplan-Meier estimate of recurrence-free survival in tamoxifen-treated ER-positive tumors. **(e) **Kaplan-Meier estimate of recurrence-free survival in untreated ER-positive tumors. HR, hazard ratio.

The aim of the present study was to evaluate the potential role of HMG-CoAR expression in predicting response to tamoxifen; subset analysis of RFS was therefore performed comparing the effect of tamoxifen versus no treatment based on HMG-CoAR expression. Figure 4a,b demonstrate that HMG-CoAR expression was associated with an improved response to tamoxifen in the entire cohort irrespective of ER status. Cox interaction analysis confirmed that HMG-CoAR expression was associated with an improved response to tamoxifen (hazard ratio = 0.5, *P *= 0.003) in the entire cohort irrespective of ER status.

Stratification according to ER and treatment status demonstrated that HMG-CoAR expression was associated with an improved RFS in all ER-positive patients, irrespective of treatment status (Figure 4c); this was confirmed by mutltivariate Cox regression analysis whereby HMG-CoAR expression was an independent positive prognostic factor in ER-positive patients (hazard ratio = 0.65, 95% confidence interval = 0.45 to 0.96, *P *= 0.029) (Table 3). This effect was maintained when tamoxifen-treated ER-positive patients were examined separately (Figure 4d). HMG-CoAR expression was not associated with a prolonged RFS in untreated ER-positive patients (Figure 4e), however, suggesting that the HMG-CoAR status may predict the tamoxifen response.

**Figure 5:**
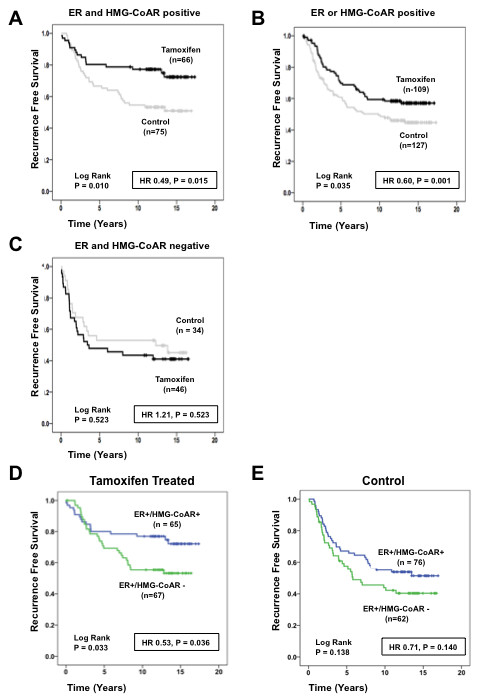
**Relationship between estrogen receptor, HMG-CoAR and tamoxifen response in premenopausal breast cancer**. **(a) **Kaplan-Meier estimate of recurrence-free survival based on tamoxifen treatment in estrogen receptor (ER)-positive and 3-hydroxy-3-methylglutharyl-coenzyme A reductase (HMG-CoAR)-positive tumors (*n *= 141). **(b) **Kaplan-Meier estimate of recurrence-free survival based on tamoxifen treatment in ER-positive or HMG-CoAR-positive tumors (*n *= 236). **(c) **Kaplan-Meier estimate of recurrence-free survival based on tamoxifen treatment in ER-negative and HMG-CoAR-negative tumors (*n *= 80). **(d) **Kaplan-Meier estimate of recurrence-free survival in tamoxifen-treated ER-positive patients based on HMG-CoAR expression. **(e) **Kaplan-Meier estimate of recurrence-free survival in untreated ER-positive patients based on HMG-CoAR expression. HR, hazard ratio.

Based on these findings, the relationship between ER and HMG-CoAR expression and tamoxifen response was examined. Three subsets were constructed: ER-negative and HMG-CoAR-negative (*n *= 80), ER-positive and HMG-CoAR-positive (*n *= 141), and ER-positive or HMG-CoAR-positive (*n *= 236). Analysis of these groups revealed that patients with ER-positive and HMG-CoAR-positive tumors had a significant response to tamoxifen (*P *= 0.010) (Figure 5a) as well as patients with ER-positive or HMG-CoAR-positive tumors (*P *= 0.035) (Figure 5b). Double-negative tumors did not respond to tamoxifen (Figure 5c). Stratification according to ER and HMG-CoAR status demonstrated that ER-positive/HMG-CoAR-positive tumors had an improved RFS compared with ER-positive/HMG-CoAR-negative tumors in the treatment arm (*P *= 0.033); this effect was lost in the control arm (*P *= 0.138), however, suggesting that HMG-CoAR predicts tamoxifen response (Figure 5d,e).

## Discussion

The aim of the present study was to evaluate HMG-CoAR expression as a predictive marker of tamoxifen response. Our results demonstrate a potentially important association with tamoxifen response both *in vitro *and using two independent breast cancer cohorts, one of which encompasses a randomized control trial of premenopausal stage II disease.

A wealth of evidence supports the role of tamoxifen in the treatment of breast cancer [25]; however, resistance to tamoxifen is a significant clinical problem - and the Oxford meta-analysis reported a relapse rate of 15%, and an 8% incidence of breast-cancer-specific mortality within 5 years of the commencement of therapy [25]. The introduction of aromatase inhibitors may lead to an improvement in postmenopausal women [26,27], but resistance to aromatase inhibitors may become a problem over time. In premenopausal women, tamoxifen remains the endocrine drug of choice and both inherent and acquired resistance may be more prevalent in this population. Tamoxifen resistance in premenopausal women is quite hard to quantify accurately as the majority of premenopausal patients receive adjuvant cytotoxic chemotherapy; however, Ryden and colleagues reported a recurrence rate of 39.7% at 10 years in the absence of adjuvant chemotherapy in the trial from which Cohort II was derived [19]. An ability to identify patients who will respond to endocrine therapy prior to commencing treatment would be extremely beneficial.

To that end, the data presented here are particularly interesting. Cox regression analysis confirmed HMG-CoAR was an independent prognostic marker in both cohorts. Cohort I was a nonrandomized cohort consisting of a mixture of premenopausal and postmenopausal patients and therefore cannot be used to validate a predictive biomarker; however, this cohort provides important information, particularly as HMG-CoAR mRNA expression was measured as a continuous variable. The relationship between HMG-CoAR protein expression and tamoxifen response was examined in Cohort II, a randomized exclusively premenopausal cohort, and a Cox interaction analysis revealed an interaction between tamoxifen treatment and HMG-CoAR expression. Subset analysis of Cohort II revealed that tumors expressing both ER and HMG-CoAR were particularly sensitive to tamoxifen, but tumors that expressed either ER or HMG-CoAR also responded to tamoxifen. In addition HMG-CoAR-positive/ER-positive patients had a significantly improved response to tamoxifen compared with HMG-CoAR-negative/ER-positive patients.

In contrast to previous studies by our group, none of the tumors expressed high levels (3+) of HMG-CoAR. This difference could potentially be explained by the fact that Cohort II was an exclusively premenopausal cohort with stage II breast cancer, which also explains the positive association with lymph node status seen in the present study. In our two previous breast cancer studies we demonstrated an inverse relationship between tumor size and HMG-CoAR [13,14], and therefore the absence of small stage I tumors from Cohort II could explain the lack of tumors expressing high levels of HMG-CoAR in this study. This argument is further strengthened by the inverse relationship between HMG-CoAR mRNA expression and tumor size demonstrated in Cohort I in this study, as this cohort also included a significant number of small stage I tumors. We are unable to explain the positive association between Her2 and HMG-CoAR seen in Cohort II, but this could potentially be explained by the association with lymph-node-positivity.

Such findings raise the possibility of pharmacological interventions to increase tumor-specific HMG-CoAR expression as a potential therapeutic option for breast cancer. Statin-induced mevalonate depletion has been shown to result in an adaptive induction of HMG-CoAR expression in Chinese hamster ovary cells [28] and MCF7 breast cancer cells [29]. Treatment of MCF7 cells with mevastatin resulted in a 10-fold to 15-fold induction of HMG-CoAR activity in association with a 2.5-fold to 3.5-fold induction of HMG-CoA reductase mRNA expression [29], suggesting that treatment with statins may increase tumor-specific HMG-CoAR expression *in vivo*; however, this suggestion remains to be fully elucidated. Given our findings that increased levels of HMG-CoAR expression are associated with an improved response to tamoxifen in ER-positive tumors, a combination of tamoxifen and statins may be a new therapeutic option. Further studies, however, are required to investigate the value of HMG-CoAR expression as a predictive marker of response to statin treatment.

Despite an ever-growing body of literature describing the anti-neoplastic properties of statins, epidemiologic data regarding their preventive effect against cancer in general - and breast cancer in particular - remain inconclusive [7,9,30-32]. In the adjuvant setting, a recent preoperative window trial of ductal carcinoma *in situ *and stage I breast cancer was the first to demonstrate that statins can inhibit proliferation and increase apoptosis *in vivo *[33], raising the possibility that the combination of statins and well-established chemotherapeutic and endocrine agents may be an option. A synergism between statins and trastuzumab, rapamycin and epirubicin has been demonstrated in breast cancer cell lines [34]; however, a synergistic relationship between tamoxifen and statins has yet to be investigated.

## Conclusions

These data describe HMG-CoAR as a significant predictor of tamoxifen response in premenopausal breast cancer patients with both ER-positive and ER-negative tumors. Using a cohort of patients who had participated in a randomized control trial with long-term follow-up, we have demonstrated that tumor-specific HMG-CoAR expression predicts response to tamoxifen. Tumors that express both ER and HMG-CoAR had an excellent response to tamoxifen, but tumors that express ER or HMG-CoAR also respond to tamoxifen. These findings suggest that the combination of tamoxifen and statins may be a viable and well-tolerated therapeutic option for a subset of breast cancer patients, which warrants further investigation.

## Abbreviations

ER: estrogen receptor; HMG-CoAR: 3-hydroxy-3-methylglutharyl-coenzyme A reductase; MTT: 3-(4,5-dimethylthiazol-2-yl)-2,5-diphenyltetrazolium bromide; PBS: phosphate-buffered saline; PCR: polymerase chain reaction; OS: overall survival; PR: progesterone receptor; RFS: recurrence-free survival; TMA: tissue microarray.

## Competing interests

DJB, KJ, MU and FP hold pending intellectual property in relation to HMG-CoAR as a prognostic biomarker in epithelial ovarian cancer and a predictive biomarker in tamoxifen-treated breast cancer. The remaining authors declare that they have no competing interests.

## Authors' contributions

DJB conceived the study, performed statistical analysis and drafted the manuscript. HL performed western blot analysis qRT-PCR, and the MTT assay. DP'OC conceived the study and drafted the manuscript. SB conceived the study and drafted the manuscript. MU conceived the study and drafted the manuscript. WMG conceived the study and drafted the manuscript. FP conceived the study and drafted the manuscript. RCM provided statistical analysis. LR performed statistical analysis and drafted the manuscript. KJ conceived the study, constructed the tissue microrarrays, performed and analyzed immunohistochemistry, and drafted the manuscript.
